# Strengthening Resilience and Mental Well-Being Through the Support4Resilience Toolbox for Leaders in Older Adult Care: Protocol for a Cross-Country Mixed Methods Study (Support4Resilience)

**DOI:** 10.2196/73701

**Published:** 2026-07-20

**Authors:** Hilda Bø Lyng, Cecilie Haraldseid-Driftland, Daniel Adrian Lungu, Petter Viksveen, Kristin Akerjordet, Malin Knutsen Glette, Eline Ree, Veslemøy Guise, Annie Haver, Inger Johanne Bergerød, Eila Kankaanpaa, Georgia M Kapitsaki, Andreas Chatzittofis, Louise A Ellis, Roland Bal, Florin Tibu, Juana Maria Delgado-Saborit, Anna Tolosa, Paola Cantarelli, Federico Vola, Mari Lahti, Henk Parmentier, Maren Kristine Raknes Sogstad, Carsten Engel, Charles Vincent, Jeffrey Braithwaite, Holger Pfaff, Siri Wiig

**Affiliations:** 1 Faculty of Health Sciences SHARE-Centre for Resilience in Healthcare University of Stavanger Stavanger Norway; 2 Department of Health and Caring Sciences Western Norway University of Applied Sciences Haugesund Norway; 3 Department of Health and Social Management University of Eastern Finland Kuopio Finland; 4 Department of Computer Science University of Cyprus Aglantzia Cyprus; 5 Medical School University of Cyprus Nicosia Cyprus; 6 Australian Institute of Health Innovation Centre for Healthcare Resilience and Implementation Science Macquarie University Sydney Australia; 7 Erasmus School of Healthcare Policy & Management Erasmus University Rotterdam Rotterdam The Netherlands; 8 Faculty of Medicine and Biological Sciences Stefan cel Mare University of Suceava Suceava Romania; 9 Department of Medicine Universitat Jaume I Castellón de la Plana Spain; 10 Fundacion, Universitat Jaume I-Empresa Castello de la Plana Spain; 11 Management and Healthcare Lab Institute of Management Scuola Superiore Sant’Anna Pisa Italy; 12 Fondazione Casa Cardinale Maffi ONLUS Cecina Italy; 13 Health and Well-being Turku University of Applied Science Turku Finland; 14 European Forum for Primary Care Utrecht The Netherlands; 15 Norwegian University of Science and Technology Gjøvik Norway; 16 The International Society for Quality in Healthcare Dublin Ireland; 17 Department of Experimental Psychology University of Oxford Oxford United Kingdom; 18 Centre for Health Services Research Cologne University of Cologne Cologne Germany

**Keywords:** organizational resilience, individual resilience, burnout, informal caregivers, health care workers, mental well-being, older adult care, cross-country study, leadership, Support4Resilience

## Abstract

**Background:**

Older adult care systems face severe workforce shortages, rising demands, and high levels of stress and burnout, undermining the quality of care and organizational resilience. Support4Resilience (S4R, 2024-2028) aims to improve working conditions and mental well-being by equipping leaders with an evidence-based, organizational-level intervention. The project develops and evaluates a digital S4R Toolbox consisting of 3 tools: (1) mapping and identification (MAP); (2) reflection and education (IMPROVE); and (3) reorganization (REMOVE).

**Objective:**

The project aims to strengthen resilience and mental well-being among health care workers and informal caregivers in older adult care across Europe and Australia through the development and implementation of the digital S4R Toolbox. Secondary objectives are identifying determinants of resilience and mental well-being across diverse contexts; exploring needs and perspectives that inform successful adaptation to changing working conditions and ethical challenges; designing the S4R Toolbox; evaluating its relevance, effectiveness, and cost-effectiveness across health care systems; advancing theory on the relationship among individual resilience, organizational resilience, and leadership; and producing research-based recommendations and interventions through the open-access S4R Resource Bank.

**Methods:**

S4R applies an exploratory, longitudinal, mixed-methods co-design approach across 4 phases. The input phase gathers evidence through literature reviews, context mapping, and qualitative and quantitative data collection in 7 countries. The co-design and prototype testing phase involves developing the S4R Toolbox and conducting pilot testing. The implementation, evaluation, and finalization phase includes a 1-year implementation period, followed by process, effectiveness, and cost-effectiveness evaluations and final refinement of the Toolbox. The output phase disseminates the results through the open-access S4R Resource Bank.

**Results:**

The project has achieved substantial early progress, including 5 literature reviews, completed and published context mapping, and comprehensive data collection involving health care workers, leaders, and informal caregivers in 7 countries. Toolbox development is well advanced, and pilot testing has been completed.

**Conclusions:**

S4R will deliver a research-based digital Toolbox that supports leaders in strengthening the resilience and mental well-being of health care workers and informal caregivers in older adult care. By integrating the perspectives and experiences of leaders, health care workers, and informal caregivers, identifying resilience factors, and developing theory-informed, cost-effective interventions, S4R will provide actionable resources through an open-access platform, contributing to more resilient older adult care systems.

**Trial Registration:**

ClinicalTrials.gov NCT07504042; https://clinicaltrials.gov/ct2/show/NCT07504042

**International Registered Report Identifier (IRRID):**

DERR1-10.2196/73701

## Introduction

### The Problem

Older adult care experiences labor shortages, a lack of qualified health care workers, and a major mismatch between health care service capacity and demand, leading to stress, burnout, and reduced resilience and mental well-being among health care workers, leaders, and informal caregivers (individuals, often family members, who provide some form of unpaid, ongoing care in activities of daily living for older adults) [1-4]. These issues challenge resilient performance and can compromise care quality, further hindering workforce recruitment and retention. This exacerbates the burden on formal and informal caregivers and increases the risk of patient harm. Urgent action is needed to address these problems, especially in older adult care, where the workforce tends to be insufficiently skilled and leaders often have limited training. Contextual risks vary significantly across geographical settings and among community-based and institutional services in different countries [1,5,6].

### A Research-Based Solution

The Support4Resilience (S4R) project (2024-2028) aims to strengthen working conditions, service quality, and patient safety in older adult care by supporting leaders in reframing work environments and enhancing organizational structures. To meet the growing demands of older adult care, health care leaders need research-based, effective, and cost-effective tools to foster resilient performance and mental well-being by enhancing the capacity of both individuals and organizations to adapt to changing conditions and new requirements [7-9]. Building such adaptive capacity requires health care leaders to support individuals and organizations in coping with challenges, reframing practices, and innovating solutions [10].

Sustainable improvements in mental well-being call for a shift from individual-focused interventions to organizational approaches. Organizational-level interventions have been found to produce more sustainable and positive effects than interventions targeted at the individual level [11]. Leaders therefore need accessible, scalable interventions that address risks to well-being at the organizational, team, and individual levels while embedding mental well-being and resilience as integral components of the workplace.

### The Project’s Ambition

**Figure 1:**
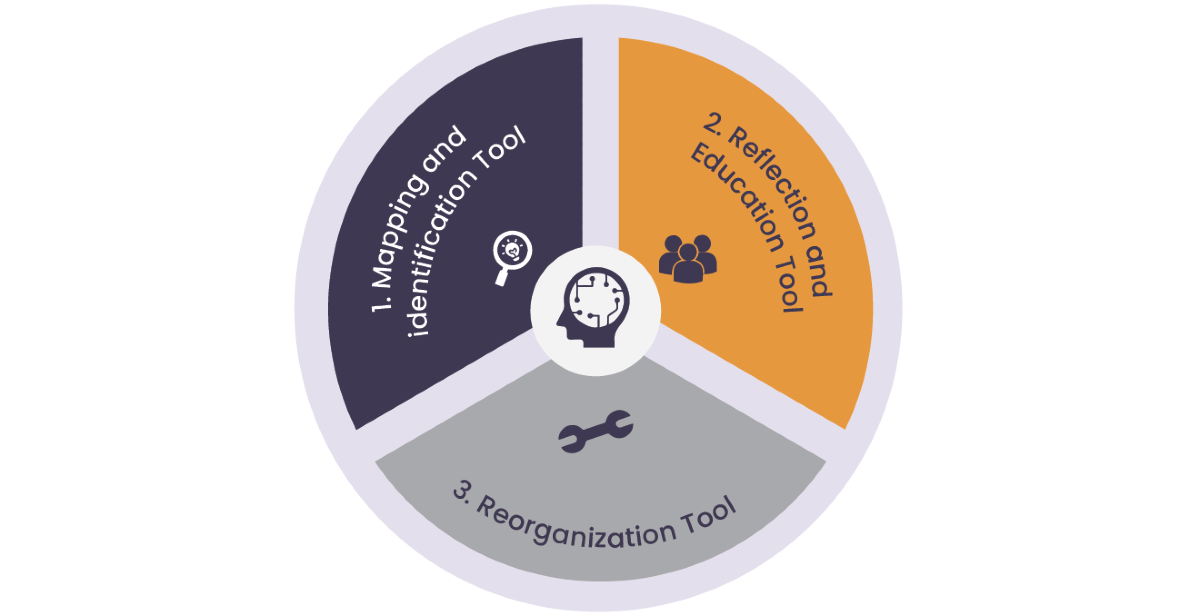
Support4Resilience Toolbox.

The S4R project will advance the current state of the art by developing, implementing, and evaluating a research-based digital S4R Toolbox to support health care leaders in promoting resilient performance and mental well-being among health care workers and informal caregivers in European and Australian older adult care. The S4R Toolbox will be an online platform that provides leaders with effective individual and collaborative tools designed to support leadership action. Its focus will be on creating a sound system in which leaders are competent and able to adapt to workforce needs and suggestions, with the potential to improve working conditions, mental well-being, resilient performance, and reduce stress and burnout. The project also aims to build an understanding of how to adapt to change, respond to adverse events, and innovate under increasingly demanding circumstances. The ambition is to generate an “exponential” effect, whereby implementing the S4R Toolbox through health care leaders improves conditions for many health care workers and informal caregivers in older adult care. The S4R project envisages a digital toolbox with 3 separate tools (see Figure 1):

- A Mapping and Identification Tool that will provide leaders with a holistic understanding of the current status of the organization by integrating the perspectives of health care workers and informal caregivers.
- A Reflection and Education Tool that will provide a contextual understanding of “work-as-done” rather than “work-as-imagined” and educational components to identify successful adaptations and solutions for reducing capacity-demand gaps.
- A Reorganization Tool that will be designed to support reframing by identifying which practices to preserve, implement, or de-implement, resulting in detailed action plans (see Figure 1).

### Objectives

Through the S4R digital Toolbox, the primary objective of the project is to support leaders in older adult care across Europe and Australia in improving resilience and mental well-being among health care workers and informal caregivers by integrating their perspectives and suggestions for improving working conditions. For the secondary objectives and research questions, see Table 1.

**Table 1:** Secondary objectives and research questions.

Secondary objectives	Research questions
To map and identify factors associated with resilience and mental well-being among health care workers, informal caregivers, and leaders in older adult care across 6 European countries and Australia, and to integrate contextual factors, geographical settings, gender perspectives, and capacity-demand challenges.	How can resilience and mental well-being among health care workers, informal caregivers, and leaders in older adult care across different health care systems be understood, and what factors are important for promoting resilience and mental well-being in older adult care services?
To explore the needs and perspectives of health care workers and informal caregivers to support successful adaptation to changing working conditions, ethical dilemmas, and adverse events, and to identify potential solutions.	What needs and perspectives of health care workers and informal caregivers support successful adaptation to changing working conditions? What successful adaptations can be identified as solutions to challenges and changing working conditions across different older adult care settings?
To develop the digital S4R^a^ Toolbox for leaders to promote resilient performance and mental well-being based on the characteristics, needs, involvement, and improvement suggestions of health care workers and informal caregivers.	How can a leadership tool be designed and developed to promote resilience and mental well-being among health care workers and informal caregivers?
To implement and evaluate the use, relevance, effectiveness, and cost-effectiveness of the S4R Toolbox across different health care systems in 6 European countries and Australia.	What are the relevance, effectiveness, and cost-effectiveness of the S4R Toolbox for improving the resilience and mental well-being of health care workers?
To develop a theoretical framework describing the relationships among individual resilience, organizational resilience, mental well-being, and the role of leaders in promoting these outcomes.	How can theories of mental well-being and individual and organizational resilience be integrated? What role do leaders play in promoting resilience and mental well-being?
To develop and disseminate empirically grounded, context-sensitive recommendations and cost-effective interventions for policy makers and leaders through the open-access S4R Resource Bank.	What lessons can be learned from the S4R intervention, and how can they be used by policy makers to improve older adult care?

^a^S4R: Support4Resilience.

## Methods

### Study Design and Phases

**Figure 2:**
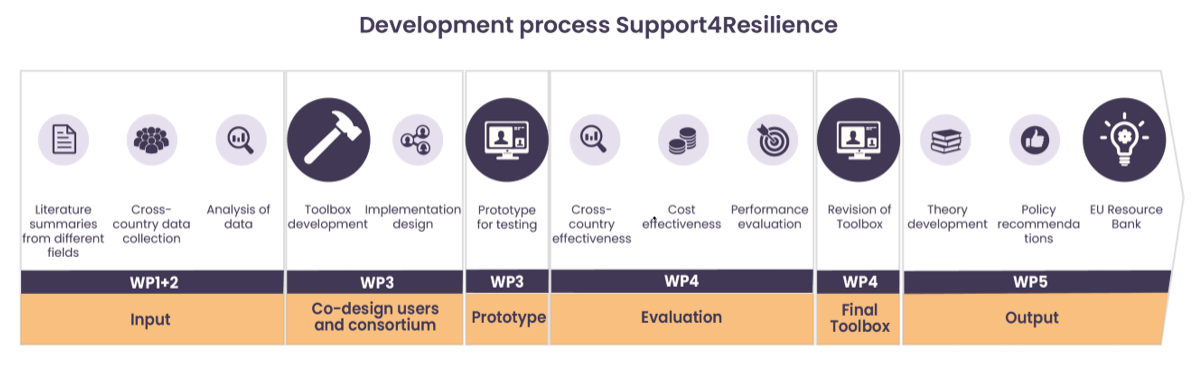
Final Support4Resilience (S4R) development process. EU: European Union; WP: work package.

The S4R project applies an exploratory, longitudinal, mixed-methods co-design methodology to achieve the intended project results, outcomes, and impacts [12] (see Figure 2). It comprises 4 phases and 5 main work packages (WPs):

- Input phase (WP1 and WP2)
- Co-design of the S4R Toolbox and prototype testing phase (WP3)
- Implementation, evaluation, and finalization phase (WP4)
- Output phase (WP5)

The choice of methodology is based on and aligned with the project objectives of developing, implementing, and evaluating a research-based toolbox to support leaders. The process will be based on the Medical Research Council’s (MRC) guidelines [13] for developing, implementing, and evaluating complex interventions to improve health care. This framework emphasizes the importance of context, including adaptation to local settings, to ensure success in practice.

**Figure 3:**
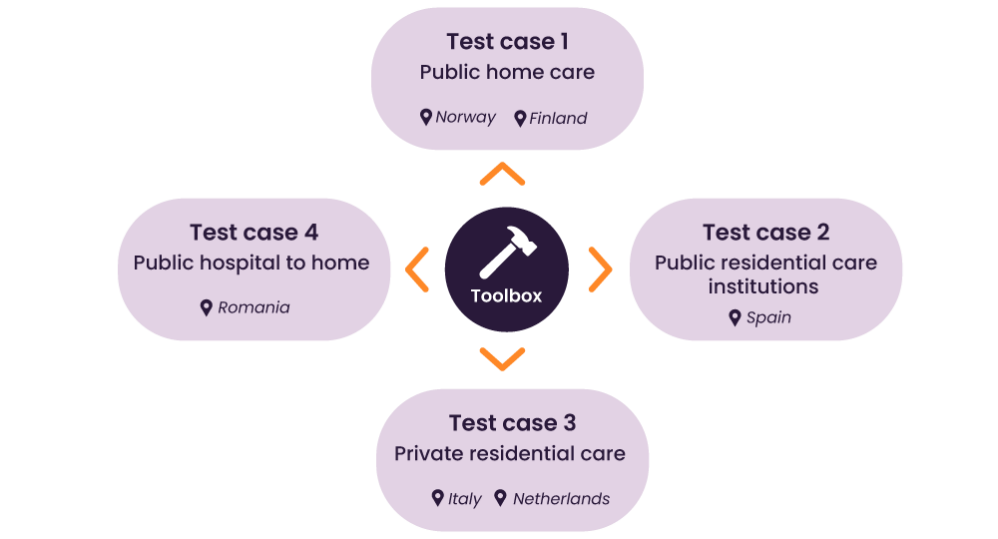
Support4Resilience (S4R) test cases in different countries.

The countries included in the project were selected because they organize health care services differently, allowing the S4R Toolbox to be tested under varying conditions. As such, the objective is not to compare outcomes across countries but rather to ensure a positive impact (measurable, observable, or experienced improvements in the resilience and mental well-being of health care workers in older adult care, supported by strengthened leadership practices and improved working conditions) in each setting. The test sites in each country will be recruited to contribute to data collection (interviews and surveys) during the input phase (pretest), toolbox testing, and data collection (interviews, observations, and surveys to enable process evaluation and assess the effectiveness and cost-effectiveness of the toolbox) during the implementation and evaluation phase (posttest). The test sites will include units with a total sample size of 250-500 participants in each country, consisting of health care workers, health care leaders, and informal caregivers. This will provide the basis for pre- to posttest comparisons for test cases 2-4 (see Figure 3). In addition, test case 1 (Norway and Finland) will participate in a cluster randomized controlled trial (cRCT) to assess the effectiveness and cost-effectiveness of the intervention (see Figure 3 for the different test cases).

Test case 1 will be carried out in Finland and Norway, 2 countries with the most similar older adult care systems (eg, number of older adults receiving care services and home care services), where public home care services are common and are the responsibility of municipalities or counties [14]. Data will be collected, and the S4R Toolbox will be tested in public home care services across a variety of geographical locations (including rural areas), municipalities, and travel distances for health care workers and informal caregivers.

Test case 2 will take place in public residential care institutions and residential care institutions receiving public funding in Spain. Data will be collected, and the S4R Toolbox will be tested in the context of public residential care services. This context is common in several European countries that provide publicly funded residential care services for older adults.

Test case 3 focuses on private residential care in Italy, the Netherlands, and Australia. In Italy, the S4R Toolbox will be tested in a large facility with 450 employees. In the Netherlands and Australia, the S4R Toolbox will be tested in private residential care networks because of the structure of their health care systems. Australia will conduct an independent evaluation because of its complementary Australian funding.

Test case 4 represents the part of Europe where home care services and residential care homes are largely absent. In these settings, families provide care outside hospitals [14]. For this purpose, data will be collected in Romania, and the S4R Toolbox will be tested in an older adult care context representing the hospital-to-home interface. Health care services for older adults in Romania are provided primarily by hospitals, whereas public services to support patients in their homes are limited. Consequently, home care is typically provided by patients’ families. Recruiting a public hospital will therefore enable the project to investigate relevant aspects of older adult care within these types of health care systems.

### Study Phases

#### Input Phase (WP1 and WP2)

**Figure 4:**
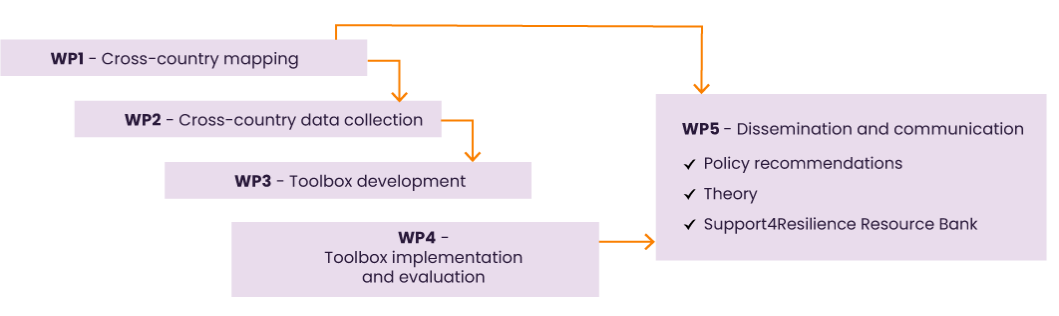
Flow of data in the Support4Resilience (S4R) project. WP: work package.

The input phase includes WP1 (preparation for data collection) and WP2 (cross-country data collection) and spans months 1-17 (see Figures 2 and 4). This phase will map and identify factors influencing resilience and mental well-being among health care workers, informal caregivers, and leaders in older adult care across 6 European countries (Finland, Italy, the Netherlands, Norway, Romania, and Spain) and Australia. It aims to provide a holistic understanding of the key factors that support leaders in older adult care. Furthermore, it will explore the needs and perspectives of health care workers and informal caregivers to enable successful adaptation to changing work conditions, ethical dilemmas, and adverse events and to identify potential solutions. The main activities in this phase include the following: (1) literature reviews; (2) contextual mapping of health care systems; (3) mapping of resilience and mental well-being factors among health care workers, informal caregivers, and leaders; and (4) exploration of health care workers’ and informal caregivers’ perspectives.

#### Literature Reviews

We will supplement the existing research literature [15-17] by conducting 3 reviews on the following topics: (1) factors supporting individual and organizational resilience and the relationship between individual and organizational resilience [18]; (2) factors and interventions supporting the mental well-being of older adult care workers [19]; and (3) the effectiveness and cost-effectiveness of organizational management interventions for promoting mental well-being in older adult care [20].

First, the findings will enable the development of a cross-disciplinary table of definitions, measures, psychometric properties, and identified research gaps, which will be combined into novel, holistic theoretical concepts and used to develop a Resilience Capacity Scale (RCS). Second, the relationships between resilience, mental well-being, and economically relevant outcomes, such as sick leave, disability, turnover, burnout, and productivity, will be mapped. Results from the literature review on intervention and implementation design in older adult care will inform the S4R intervention design, including final decisions on the selection of instruments for quantitative and cost-effectiveness analyses. Third, the findings will inform the content of questionnaires and interview guides for collecting data from leaders, health care workers, and informal caregivers.

#### Contextual Mapping of Health Care Systems

The organization of older adult care differs across European countries and Australia. The project will map a broad range of external and internal contextual factors that may influence the research and implementation of interventions to improve resilience, mental well-being, quality, and patient safety in older adult care services. Information on external contextual factors will be collected through publicly available sources, including national, regional, and local government websites, as well as from relevant authorities. Internal contextual factors will be collected from the recruited sites. The context mapping tool is based on an existing tool designed for mapping older adult care contexts across different countries [21,22] and is anchored in the Consolidated Framework for Implementation Research developed by Damschroder et al [23].

#### Resilience and Mental Well-Being Factors Among Health Care Workers, Informal Caregivers, and Leaders

An online survey will be conducted in the 6 European partner countries and Australia. WP2 aims to map resilience and mental well-being factors and explore their associations across 7 health care systems. A total sample of 2500-3000 participants (minimum of 300 leaders, 1000 informal caregivers, and 1200 health care workers) will ensure:

- Adequate statistical power for multivariable regression (at least for informal caregivers and health care workers)
- Sufficient representation of health care workers, leaders, and informal caregivers
- Reliable estimation of psychometric properties, including those of the new RCS.

WP2 is descriptive and exploratory; multivariable regression models will be used to explore associations among resilience, mental well-being, job characteristics, and demographic factors and to inform instrument development and intervention design. These analyses are not intended for confirmatory hypothesis testing. Consequently, no formal power calculation was conducted for WP2. Instead, the sample size was determined based on feasibility, stakeholder representation, and the requirements for psychometric evaluation. Moreover, the limited availability of leaders in older adult care must be taken into account.

The participating countries represent highly diverse health care systems (see Figure 3). Informants will represent different genders and age groups, and health care workers and health care leaders will have diverse professional backgrounds and experiences. The survey will be available in the official language of each country. Informants at the recruited sites will be recruited through leaders and the use of QR codes, whereas informal caregivers will be recruited through the participating sites as well as through additional networks and social media. The baseline assessment will measure mental well-being, resilience capacities, individual resilience, patient safety perceptions, job demands and resources, work engagement, job satisfaction, leadership support, burnout, intention to quit, sick leave, and health-related quality of life at the recruited sites in each country. The instruments used for health care workers and health care leaders are the Short Warwick-Edinburgh Mental Well-Being Scale (SWEMWBS) [24], the RCS (in development) [25], the Copenhagen Burnout Inventory [26], the Motivation at Work Questionnaire intention-to-turnover measure [27], and the Self-Assessed Health measure [28]. For informal caregivers, the Family Involvement in Care Questionnaire [29] and the Caregiver Self-Assessment Questionnaire [30] will be used (see Multimedia Appendix 1).

#### Translation Process

The project spans 7 countries with diverse languages (Finnish, Norwegian, Italian, Dutch, Romanian, Spanish, and English). Where validated translations of the instruments already exist, these will be used in the corresponding country. For tools that are not yet available in 1 or more study languages (including the newly developed RCS), we will follow internationally recognized procedures for translation and cultural adaptation [31]. Specifically, the process will include translation into the target language by multiple translators, resolution of discrepancies, expert committee review (researchers from the participating countries), cognitive testing, and piloting with a small sample, with the exception of the back-translation stage of the framework described by Beaton et al [31]. The translation process will be documented in a report.

This approach ensures that the measurement tools are conceptually equivalent across settings while recognizing that the project does not aim for strict cross-country comparability but instead focuses on within-country validity and the context-sensitive evaluation of the S4R Toolbox.

Given the heterogeneity of health care systems across the participating countries, the purpose of these measures is not to enable cross-country score comparisons but rather to provide robust, contextually appropriate assessments of resilience and mental well-being within each national system.

#### Quantitative Analyses

Analysis in WP2 will describe the distribution of mental well-being, resilience capacities, burnout, intention to quit, support, and other relevant variables across countries. The analysis plan includes the following (with ad hoc customizations depending on the stakeholder group: health care workers, leaders, or informal caregivers):

- Descriptive analyses (means, SDs, and proportions) will be produced for each stakeholder group within each country.
- Internal consistency of multiitem instruments will be assessed using Cronbach α.
- Exploratory factor analysis will be conducted in a development subsample, and confirmatory factor analysis will be conducted in an independent holdout subsample within the baseline data to confirm the factor structure of the RCS.
- Multivariable regression models will examine associations among resilience, mental well-being, job characteristics, and demographic factors.

These analyses will provide contextual understanding and empirical input for the development of the S4R Toolbox.

#### RCS Development and Validation

The RCS was developed to capture organizational resilience capacities relevant to older adult care (eg, adaptive capacity, learning, coordination, and supportive structures), grounded in resilience-in-health care theory and organizational approaches to workforce well-being. WP1 covers theoretical specification, item generation, expert review (content validity), translation and cultural adaptation, and cognitive testing and piloting before field deployment.

Psychometric evaluation will be conducted using the WP2 baseline dataset. To avoid circularity, we will use internal cross-validation: exploratory factor analysis will be performed in a development subsample, and confirmatory factor analysis will be conducted in a separate holdout subsample. Reliability (eg, internal consistency) and construct validity will also be assessed. The final scoring algorithm and measurement model will be specified (locked) before the WP4 effectiveness analyses. In WP4, the RCS will be used as a secondary outcome to assess changes in organizational resilience capacities. If minor refinements are made after baseline validation, the WP4 analyses will use the locked version, and sensitivity analyses will be reported where relevant.

#### Handling of Missing Data

Patterns and mechanisms of missing data will be examined. Missing data will be handled using multiple imputation under a missing-at-random assumption. The imputation model will include all analysis variables (primary and secondary outcomes, baseline covariates, treatment group, and cluster identifiers), as well as auxiliary variables associated with missingness, to improve the plausibility of the missing-at-random assumption. Approximately 20-50 imputations will be generated, with the final number guided by the proportion of missing information.

Patterns of missingness will be examined to distinguish monotone (eg, dropout after baseline) and nonmonotone patterns, and appropriate imputation methods will be applied accordingly. For the cRCT, potential differential attrition between the intervention and control groups and across clusters will be assessed and reflected in the imputation model.

Sensitivity analyses will compare imputed results with complete-case analyses and alternative assumptions (eg, pattern-mixture approaches) to evaluate robustness.

#### Exploration of Health Care Workers’ and Informal Caregivers’ Perspectives

Qualitative data collection will take place at the recruited sites in the same 6 European countries and Australia. The data will be collected through semistructured interviews with leaders, health care workers, and informal caregivers to explore their diverse challenges in everyday tasks and care situations, types of ethical stress, adverse events, situational support, needed adaptations, solutions to improve working and caring conditions, how they integrate and experience patient and user involvement, and the role of stakeholders in resilient service performance and in improving involvement. This will include 3-5 focus groups with leaders, 3-5 focus groups with health care workers, and 10 individual interviews with informal caregivers in each participating country (see Multimedia Appendix 2).

The interviews will be conducted in the informants’ native languages to allow for rich data and nuanced insights. The qualitative data will be analyzed using inductive thematic analysis [32], in which the data are analyzed inductively to allow themes and insights to emerge. The findings will be reported by the researchers in each country using a predefined, standardized template in English. This approach will allow for cross-country analysis of patterns across cases, as well as integration and comparison across health care systems and informant groups. Informants will be recruited in collaboration with the participating sites in all countries. Interviews will take place at the workplace for health care workers and leaders and at the preferred location of the informal caregivers. Focus groups will last approximately 2 hours each, whereas individual interviews will last approximately 1 hour each.

### Co-Design of the S4R Toolbox and Prototype Testing Phase

#### Overview

The second phase (WP3) will lead to the development of a digital S4R Toolbox for leaders to promote resilient performance and mental well-being among health care workers and informal caregivers. WP3 begins in month 5 and continues through month 30. The features and content of the S4R Toolbox will be developed based on findings from the first input phase, systematic literature reviews, and cross-country qualitative and quantitative data collection. Cross-country data from diverse settings will be used to provide an understanding of factors and challenges that are relevant across settings (residential care homes, home care services, and the hospital-to-home interface) and target groups (leaders, health care workers, and informal caregivers), ensuring that the S4R Toolbox provides value across the included older adult care settings. The development phase applies an iterative co-design methodology using an Agile software development process [33], in which a development team continuously develops the content and features of the S4R Toolbox by organizing 8 collaborative workshops (6 national and 2 transnational) with a co-design panel consisting of consortium partners and collaborators, as well as representatives of the target groups (3-5 health care leaders, 3-5 health care workers, and 3-5 informal caregivers), to ensure relevance and usability.

The S4R Toolbox includes 3 tools: (1) Mapping and Identification Tool (to increase awareness), (2) Reflection and Education Tool (to increase understanding), and (3) Reorganization Tool (to support reframing; Figure 1). While the S4R Toolbox is intended for leaders, the tools rely on the involvement of different stakeholders, including health care workers and informal caregivers, to ensure collaboration across organizational levels and stakeholder groups. All test sites will implement and evaluate the S4R Toolbox through a 1-year intervention program. The S4R Toolbox aims to bring about organizational- and system-level changes through leaders as change agents to improve working conditions for frontline health care workers and informal caregivers. Accordingly, the intervention requires sufficient time for these changes to take place.

#### Tool 1: The Mapping and Identification Tool

This tool will provide several reports and graphs based on data collected from different stakeholders (ie, informal caregivers, frontline leaders, and health care workers) to enable leaders to gain a comprehensive overview of the current status of their organization. The main input will be gathered through questionnaire responses from the involved stakeholders. The questionnaire will be intentionally developed for this tool and will pertain to 3 main areas identified through previous literature reviews: Supporting the Worker, Improving Working Conditions, and Improving Work Culture. The questions will be answered on a 5-point Likert scale and will address areas such as how well the unit fosters learning and professional growth, creates a balance between control and autonomy, develops a shared understanding of professional practice, and promotes communication and positive relationships at work.

Suitable graphs (eg, histograms and bar graphs) will be used to visualize the results, while natural language processing techniques will be used to facilitate leaders’ exploration of the findings. Presenting the data in multiple formats will further enhance the user-friendliness of the tool. The purpose is to provide leaders with a quick, easy-to-use overview of the current status of the unit as a basis for using tool 2.

#### Tool 2: The Reflection and Education Tool

Building on the same 3 themes addressed in tool 1, this tool is structured into 3 distinct learning modules: Supporting the Worker, Improving Work Conditions, and Improving Work Culture. Based on the scores obtained in tool 1, leaders are encouraged to begin with the module in which their unit demonstrates the strongest performance. Each module incorporates short informational videos, reflective questions, and brief learning scenarios depicting everyday work situations relevant to the thematic area. Through these activities, leaders, together with their staff, are expected to identify existing strengths and the underlying reasons for them.

The overarching aim is to enhance understanding of how work is carried out within the unit, identify resilient and effective practices, and promote improvement by learning from what works well. Once leaders have gained insight into successful practices, they are encouraged to translate this knowledge into targeted actions to address areas that require further development.

#### Tool 3: The Reorganization Tool

This tool addresses a well-known challenge in improvement work: the tendency to continually add new tasks and procedures without removing outdated ones. Over time, this can lead to overlapping practices and reduced system coherence. The aim of tool 3 is therefore to help units identify and safely discontinue practices that no longer add value.

The tool will include a function for gathering input from leaders, staff, and family members on activities they consider unnecessary or burdensome. Based on these insights, leaders will be provided with clear guidance and a structured action plan for removing practices in a responsible and transparent manner. The tool will also offer practical guidance on risk assessment, communication, and monitoring to ensure that discontinuation is carried out safely.

#### S4R Intervention Design Development Phase

Given the pace of work and the stressful environment of older adult care, the intervention design will be aligned with everyday work to reduce the likelihood that leaders will perceive the intervention as an additional stressor [34]. The S4R intervention will consist of 3 phases, each corresponding to 1 of the 3 tools in the S4R Toolbox. During each phase, frontline leaders will engage with different elements of the tool and participate in different activities (see Multimedia Appendix 3). The implementation of the intervention is planned to take 12 months. Six local learning collaboratives (including consortium researchers and co-researchers from the clinical setting) will be established, 1 in each European partner country, in addition to 1 principal learning collaborative comprising representatives from all 6 local learning collaboratives. Australia will have a separate learning collaborative because of time zone differences.

For each country, a designated intervention team consisting of consortium partners will be formed. The teams will be responsible for administering the local learning collaborative and coordinating the overall implementation activities throughout the intervention in their respective countries.

#### Sustainability of the S4R Toolbox

The S4R intervention design and Toolbox will be open access through the S4R Resource Bank, which includes self-directed instructions on how to implement the intervention. Furthermore, the infrastructure supporting deployment of the Toolbox will ensure server maintenance for 5 years after project completion. This period will also allow time to implement a successful exploitation strategy.

### Implementation, Evaluation, and Finalization Phase

#### Overview

The third phase (WP4) will implement and evaluate the use, relevance, effectiveness, and cost-effectiveness of the S4R Toolbox across the different health care systems involved (months 22-43; see Figures 2 and 4). Implementation of the S4R Toolbox will be carried out in line with the intervention design and the MRC guidelines [13,35-37] (see Multimedia Appendix 4). This design prioritizes the development of interventions that are both theoretically robust and practically adaptable to real-world conditions. The S4R intervention offers a coherent structure—with defined phases, tools, and recommended practices—while still enabling teams to tailor its components to their specific context, consistent with the MRC guidelines. The S4R Toolbox is intentionally flexible: although leaders are encouraged to follow the recommended structure, they may adjust the format, frequency, and integration of its elements to suit the needs and dynamics of their particular setting.

Implementation and evaluation will contribute to exploring the value of the S4R Toolbox in terms of usability, technical features, leadership support, performance, effectiveness, and cost-effectiveness, as well as provide input for the development of policy recommendations and theoretical frameworks. Evaluation of the S4R intervention will follow the study designs used in each country. Four analyses are planned for WP4: (1) process evaluation, (2) pre-post evaluation (test cases 2-4), (3) a cRCT (Norway and Finland, test case 1), and (4) cost-effectiveness evaluation.

A qualitative process evaluation will be performed [13]. Interviews with leaders, health care workers, and informal caregivers conducted in the initial phase of the intervention will be complemented by interviews conducted during and after the intervention period. Additionally, observations will be made during workshops to explore work processes and interactions among participants during implementation of the intervention. To minimize interference with implementation, we will develop standardized informational materials that will be used across all participating units. The researchers will limit their involvement to working directly with leaders through separate workshops and will not be present in the units when the Toolbox is applied in practice. This approach is intended to reduce researcher influence on everyday workflows and ensure that the Toolbox is used as intended by the local leadership. In addition, we will systematically document the contextual circumstances of each unit through context mapping and use standardized observation forms to record events or conditions that may have influenced implementation. These strategies will help differentiate between natural contextual variation and external factors that could interfere with the implementation process.

The data collected will contribute to exploring how the S4R Toolbox supports leaders, how health care workers and informal caregivers experience involvement, and whether capacity-demand challenges improve. In addition, the content of the Toolbox will be explored (eg, comprehension of the learning materials, factors important for sustainability, and levels of engagement and reflection), as well as the nonfunctional and quality aspects of the technical platform (eg, usability, tool design, and accessibility). The knowledge gained will inform further refinements to the Toolbox. Each participating site will include 3-5 focus group interviews with leaders, 3-5 focus group interviews with health care workers (nurses, physicians, and other health care workers), and 10 individual interviews with informal caregivers.

We will assess the effectiveness of the S4R Toolbox through a cRCT (test case 1), a pre-post evaluation (test cases 2-4), and a cost-effectiveness evaluation (test case 1). In both study designs, the primary outcome will be health care workers’ mental well-being, measured using the SWEMWBS. Secondary outcome measures include individual resilience, organizational resilience capacities, burnout, turnover intention, self-assessed health, and, for informal caregivers, family involvement in care and caregiver self-assessment. In addition, sick leave will be assessed using public sick leave records where available. The effectiveness, cost-effectiveness, and cRCT analyses of the S4R intervention will provide information for decision makers in organizations at both the national and European Union (EU) levels.

#### Pre-Post Evaluation (Test Cases 2-4)

Changes from baseline to postintervention will be examined using mixed-effects regression models. Individuals will be nested within organizational units, with random intercepts included for organizational units. Time will be entered as a fixed effect. Standardized effect sizes (Cohen *d*) will be calculated to aid interpretation.

For countries using a pre-post evaluation, a sample of 250-500 participants per country is sufficient to

- Detect moderate changes (effect size, *d*≈0.25-0.36) with 95% power (taking clustering into account, assuming an intraclass correlation coefficient [ICC] of 0.05).
- Estimate within-country effects using mixed-effects models.
- Explore relevant subgroup differences.

#### cRCT (Norway and Finland; Test Case 1)

The S4R Toolbox will be evaluated in a 2-arm cRCT in 2 countries (Finland and Norway). An equal allocation ratio will be used for the 2 groups: an intervention arm, in which the S4R Toolbox is implemented by health care leaders, and a control arm, in which the S4R Toolbox is not implemented by health care leaders. The cRCT will include 30 clusters with approximately 30 health care workers per cluster. Accounting for clustering (ICC=0.05), an expected 30% response rate (approximately 9 respondents per cluster), and 6 repeated measurements (baseline plus 5 follow-up assessments) with a plausible within-person correlation of 0.5-0.7, the cRCT has 95% power to detect standardized effect sizes of approximately *d*=0.28-0.32 for mental well-being. The 6 measurement time points were chosen to align with the planned phases of Toolbox use and feasible data collection windows in the participating services. This pragmatic schedule enables monitoring of early adoption and engagement, as well as whether changes are sustained over the intervention period.

The sample size calculations assumed an ICC of 0.05 and an average of approximately 9 respondents per cluster (30% response from approximately 30 eligible workers). The assumed ICC of 0.05 was selected based on previous cluster randomized trials in primary care, home care, and organizational intervention studies, in which ICCs for psychosocial and workforce-related outcomes commonly ranged from 0.01 to 0.10. Given the lack of precise prior ICC estimates for mental well-being outcomes in older adult home care services, an ICC of 0.05 was considered a conservative and appropriate planning value. This corresponds to a cluster design effect of 1.40. To assess the robustness of the findings to uncertainty in the ICC, we conducted sensitivity analyses using ICC values of 0.03, 0.08, and 0.10 (design effects of 1.24, 1.64, and 1.80, respectively). Holding all other assumptions constant, the detectable standardized effect size ranged from *d*=0.26-0.30 (ICC=0.03) to *d*=0.30-0.35 (ICC=0.08) and *d*=0.32-0.36 (ICC=0.10), compared with *d*=0.28-0.32 under the base-case ICC of 0.05. These findings indicate that the trial is sufficiently powered to detect small-to-moderate improvements in the primary outcome.

A 30% response rate was used as a conservative planning assumption for this pragmatic workforce trial in older adult care to reduce the risk of overestimating the effective sample size under real-world conditions. Participation and retention will be supported through leadership engagement, brief, mobile-friendly questionnaires, repeated reminders, and monitoring of response rates at each measurement time point.

The primary outcome measure, the SWEMWBS, will be treated as a continuous outcome. The primary analysis will follow the intention-to-treat principle at the home care service (cluster) level. Noncompliance with the intervention may attenuate the observed intervention effect. Linear mixed-effects models will be used, including random intercepts for clusters. Group (intervention vs control) will be included as a fixed effect, and SWEMWBS (mental well-being) will be the primary outcome. Other outcomes (burnout, turnover intention, caregiver burden, etc) will be analyzed as secondary end points.

Analyses will follow the intention-to-treat principle. A complier average causal effect analysis will also be conducted to account for variation in exposure to the intervention.

#### Cost-Effectiveness Analysis

Cost-effectiveness analysis will be conducted only for the cRCT study design (Norway and Finland).

The primary outcome will be SWEMWBS, with effectiveness assessed using the same methods described in the previous section.

The costs of the intervention will be based on the time spent by leaders, health care workers, and researchers implementing the S4R Toolbox. The learning collaboratives support the leaders and are therefore expected to influence the effectiveness of the Toolbox. The cost data will be based on the following:

- Minutes that health care workers use the Toolbox (data source: log files from the digital Toolbox).
- Hours that leaders and health care workers spend in meetings discussing the themes of the Toolbox and planning deimplementation or other workplace changes (data sources: learning collaborative meetings and interviews with leaders).
- Hours that leaders and researchers spend participating in local learning collaboratives (data source: notes/minutes from the learning collaboratives, including time and participants).
- The time spent by health care workers, leaders, and researchers will be valued using nationally representative wages and social security contributions.

The time horizon for the analysis is 1 year; therefore, no discounting will be applied. The analysis adopts the perspectives of 2 funders: the participating organizations, which cover the costs of leaders’ and health care workers’ time, and the S4R project funding organization (EU), which covers the costs of researchers’ support for implementing the Toolbox.

We use intention-to-treat based analyses and evaluated the incremental net monetary benefit of the Toolbox compared to no Toolbox ("practice as usual"). We evaluated incremental net monetary benefit (NMB) in comparing the alternatives. NMB is calculated by multiplying the observed effects by the decision-makers’ willingness to pay, λ, for an extra unit of effectiveness, and then subtracting the observed cost [37,38]. As the willingness to pay for one extra unit of effectiveness in SWEMWBS is unstated, we use a range of values.

As a sensitivity analysis, we will use an algorithm to convert changes in SWEMWBS scores into WELLBY (Wellbeing-Adjusted Life Year) units [39-41]. WELLBYs have been monetized and can therefore provide a monetary estimate of effectiveness. This analysis will also provide an opportunity to interpret the results of the cost-effectiveness analysis across the range of willingness-to-pay values.

To further support the robustness of our analysis, we will also conduct a cost-benefit analysis. For this analysis, we will collect data from the participating organizations (work units) on sickness absence (spells and days), staff resignations, turnover, disability pensions, and personnel survey results for the years 2023-2026. Where possible, these measures will be monetized using national recommendations, statistics, reports, and research.

The S4R project does not aim to estimate a single pooled effect across countries. The participating health care systems differ substantially in structure, governance, workforce composition, and the availability of formal older adult care services. Because of this heterogeneity, the project follows the MRC guidance for evaluating complex interventions, which explicitly recommends that evaluation designs be adapted to local feasibility and contextual conditions.

In Norway and Finland, organizational structures allow randomization of home care units, making a cRCT feasible and appropriate for evaluating both effectiveness and cost-effectiveness. In the other participating countries, randomization was not feasible because of organizational differences and the absence of comparable units. For these countries, a pre-post evaluation is methodologically appropriate and enables examination of within-country change. This hybrid approach preserves scientific rigor by using the strongest possible design where feasible while ensuring validity and contextual relevance across diverse older adult care systems.

### Output Phase

**Figure 5:**
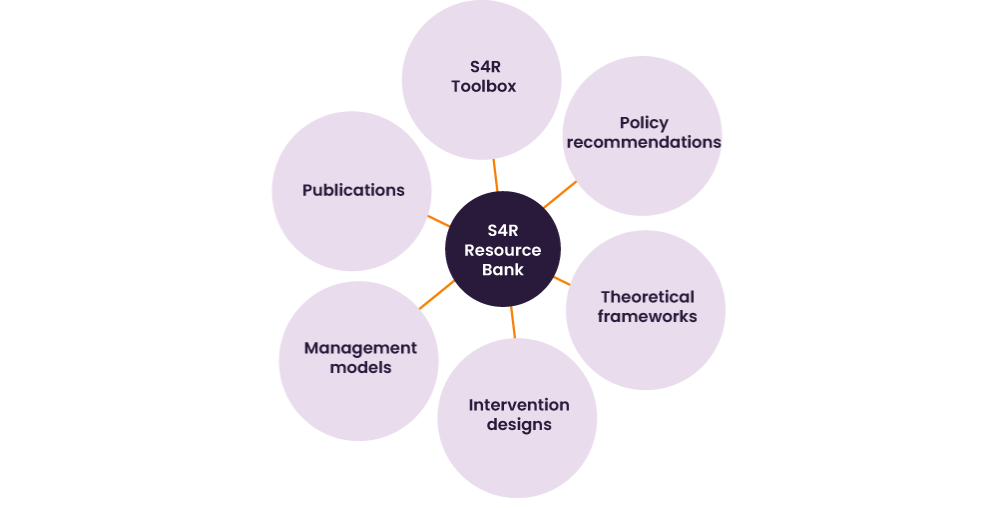
The Support4Resilience (S4R) Resource Bank.

The fourth phase (WP5) will contribute to establishing new theory to scientifically describe the relationships among individual resilience, organizational resilience, mental well-being, and the role of leaders in promoting them, as well as to developing and disseminating empirically founded, context-sensitive recommendations and cost-effective interventions for policy makers and leaders through an open-access online platform (S4R Resource Bank). Based on the data collected during the input phase, the S4R Toolbox development phase, and the implementation and evaluation of the intervention, several outputs will be developed and disseminated. First, we will provide policy makers with empirically founded, context-sensitive recommendations for older adult care to support the planning and organization of services. Second, we will develop theoretical concepts and frameworks that integrate mental well-being with individual and organizational resilience to provide a more holistic understanding. Third, an open-access, web-based S4R Resource Bank will be developed (see Figure 5). This resource will be freely available in multiple European languages, including English. The S4R Resource Bank will thereby contribute to older adult care in Europe beyond the completion of the project. The Resource Bank will include the final S4R Toolbox, resources to support implementation of the S4R Toolbox intervention, theoretical outputs, and policy recommendations. As such, it is expected to support cross-level and cross-country improvements in working conditions and strengthen mental well-being and resilience in older adult care beyond the completion of the project.

### Research Ethics

The study has been approved by the relevant research ethics bodies in Norway (reference number 881988), Finland (reference number 14/2024), Italy (reference number 22/2024), the Netherlands (reference number ETH2324-0548), Romania (reference numbers 220/25.07.2024 and 36/30.07.2024), Spain (reference number CEISH/72/2004), and Australia (reference number 52024183258818). All informants will provide written informed consent, and the data will be stored securely according to the project data management plan. The project has an ethics plan and an external ethics advisor to help maintain ethical standards throughout all phases of the project.

Data processing and sharing among consortium partners are governed by formal data processing and data sharing agreements that specify the roles of data controllers and processors, the purposes of processing, retention periods, and procedures for secure data transfer. All handling of personal data complies with the EU General Data Protection Regulation (GDPR) and applicable national legislation. Data will be pseudonymized at the source, stored on secure institutional servers with role-based access, and transferred through encrypted channels. Only deidentified datasets will be shared across countries for analysis. Deidentified datasets and metadata will be made available through the S4R Resource Bank where permitted under participant consent and national regulations. Access to individual-level data will require a data access agreement and ethics approval consistent with GDPR principles.

Participation is voluntary and based on written informed consent. Although health care workers and informal caregivers are not considered inherently vulnerable groups, potential workplace power imbalances are recognized. To mitigate these, survey responses will be collected anonymously, individual data will not be accessible to employers or managers, and participation or nonparticipation will have no consequences for employment or care relationships. Information sheets will clearly describe these safeguards and participants’ right to withdraw at any time without explanation.

## Results

### Interim Report and Study Progress

**Figure 6:**
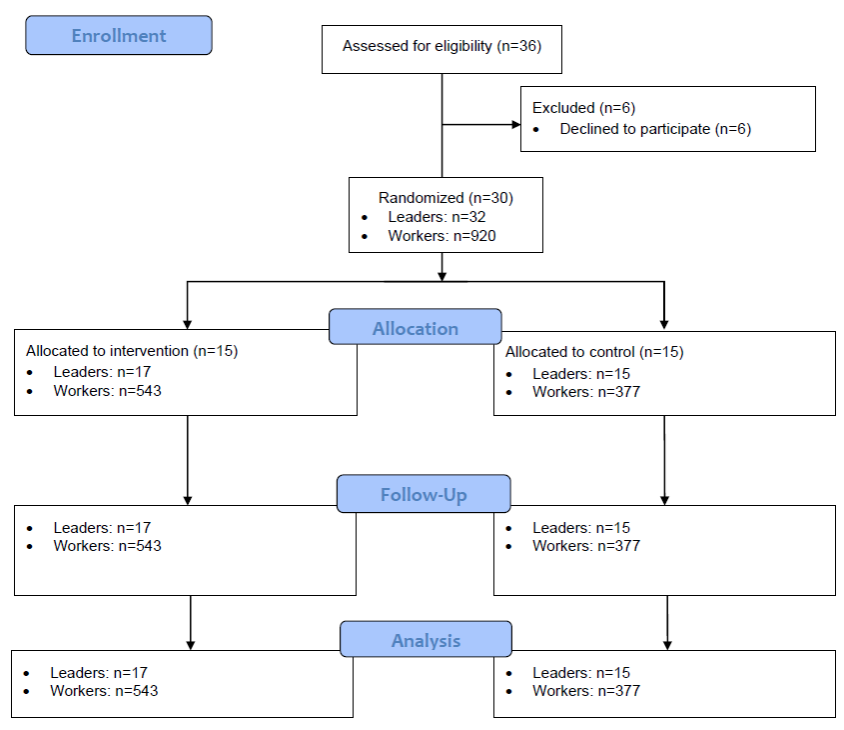
CONSORT (Consolidated Standards of Reporting Trials) flowchart.

At month 23 of the 48-month S4R project, substantial progress has been achieved in WP1, WP2, and WP3. These activities correspond to the input phase, the ongoing co-design and prototype development of the S4R Toolbox, and the newly initiated implementation phase. The planned project timeline (Gantt chart) is presented in Multimedia Appendix 5. See Figure 6 for the CONSORT (Consolidated Standards of Reporting Trials)s flowchart.

### Input Phase

WP1 has produced 5 literature reviews addressing (1) the mental well-being of older adult care workers [19], (2) the effectiveness and economic evidence of interventions promoting mental well-being and resilience [20], (3) bridging individual and organizational resilience [18], (4) leadership strategies for supporting workers’ mental well-being and individual resilience (under review), and (5) digital tools for resilience, which are in the final stages of preparation for submission. Collectively, these reviews have been instrumental in informing the development of the S4R Toolbox.

In addition, WP1 conducted a cross-country mapping study to characterize older adult care systems, associated challenges, and existing response strategies in the participating countries. The findings of this study have been published [14] and provide an important contextual foundation for the development of the S4R Toolbox and subsequent implementation activities.

WP2 has generated extensive quantitative and qualitative data examining the perspectives and roles of frontline leaders, health care workers, and informal caregivers in older adult care. Data analysis is ongoing, and several scientific publications are in preparation. These data directly inform the development and refinement of the S4R Toolbox to ensure its relevance across settings and target groups (leaders, health care workers, and informal caregivers).

### Co-Design of the S4R Toolbox and Prototype Testing Phase

WP3 focuses on the development of the S4R Toolbox and currently represents the central activity of the project. The core content and technical infrastructure of the Toolbox have been finalized, and pilot testing is underway. Preliminary feedback from participants indicates that the S4R Toolbox is perceived as relevant and useful, and insights from the pilot phase are informing final adjustments before large-scale implementation.

Findings from the input phase, the literature reviews, and the qualitative data informed the content development of the S4R Toolbox. Three modules, each with 2 subthemes, were developed. In the first round, an inductive thematic analysis of the review findings and qualitative data identified themes and subthemes for the Toolbox. The second round involved a consensus process conducted over several rounds with participation from all empirical partners to establish a shared understanding and ensure relevance across all participating settings. The modules and subthemes are as follows: the Improving Work Culture module comprises Building Positive Relationships and Facilitating Collaboration and Communication; the Supporting the Worker module comprises Fostering Professional Growth and Development and Navigating the Demands of Work and Life; and the Improving Work Conditions module comprises Balancing Management Control and Staff Autonomy and Establishing Shared Standards of Practice.

### Implementation, Evaluation, and Finalization Phase

Full implementation of the S4R Toolbox in 6 European countries (Norway, Finland, Italy, the Netherlands, Romania, and Spain) and Australia is scheduled to begin in April 2026. Preparatory work has included the development of the implementation plan (see Multimedia Appendix 4) and supporting materials for the workshop-based intervention. For the cRCT, intervention and control sites have been randomized and are ready for study initiation.

## Discussion

### Anticipated Principal Contributions

The S4R project is designed to generate new knowledge on how a research-based toolbox for leaders may strengthen organizational resilience and promote the mental well-being and individual resilience of health care workers and informal caregivers in older adult care across Europe. By integrating theory, contextual mapping, co-design approaches, toolbox development, and multicountry evaluations, the project is expected to clarify how organizational and individual resilience and mental well-being interact, and how leadership practices can act as a mediator for sustainable system transformation.

Specifically, the project addresses 3 interrelated areas. First, it advances theoretical integration by bringing together the literature on organizational resilience, individual resilience, and mental well-being—fields that have largely developed in parallel. Second, it develops and evaluates a research-based digital toolbox aimed at supporting leaders in strengthening resilience and mental well-being. Third, it generates policy-relevant evidence regarding strategies that may improve working conditions, resilience, and mental well-being across diverse older adult care systems.

### Integration of Individual and Organizational Resilience

Existing literature consistently points to a gap in research integrating individual and organizational resilience, despite increasing recognition of their interdependence [18,42,43]. While studies have documented associations among organizational conditions, work demands, work-life balance, and individual resilience, empirical evidence on how organizational strategies can systematically strengthen individual resilience remains limited [44,45].

Findings from the S4R input phase suggest a reciprocal relationship, whereby higher levels of organizational resilience may support individual resilience, and higher individual resilience may, in turn, reinforce organizational resilience [18]. In addition, findings from another S4R review highlight multilevel contributory factors (macro, meso, and micro) influencing mental well-being, underscoring the importance of extending intervention strategies beyond individual-level approaches to include organizational-level initiatives [19]. These observations are further supported by S4R review findings indicating a lack of research and rigorous evaluations of managerial and organizational interventions aimed at strengthening resilience and mental well-being [20]. Addressing this gap is essential for developing evidence-based leadership strategies that effectively promote resilience within older adult care systems.

The S4R project will further examine the nature and strength of these interactions. By synthesizing evidence from systematic reviews, empirical investigations, and evaluations, the project contributes to a more integrated conceptualization of organizational resilience that acknowledges its intersections with mental well-being and individual resilience.

### Leadership as a Mechanism for Organizational Transformation

Current research provides limited insight into how leadership simultaneously influences individual and organizational resilience [18]. The S4R intervention positions leaders as key change agents capable of shaping work environments, influencing resilient performance, and fostering supportive organizational cultures. Leaders in older adult care operate in complex and resource-constrained systems, and their role in shaping frontline working conditions is well established [9].

However, leaders often lack structured, evidence-based tools specifically designed to strengthen organizational resilience [20]. While many interventions focus on leadership training, fewer initiatives provide practical resources aimed at enhancing organizational resilience [46]. The S4R Toolbox addresses this gap by equipping leaders with actionable, research-informed strategies. Strengthening leadership capacity may therefore represent an important pathway toward improving organizational and individual resilience, as well as the mental well-being of carers [18].

### Organizational-Level Approaches to Resilience

Traditionally, resilience and well-being interventions in health care have focused predominantly on individual-level strategies [11,18,19]. Although such approaches may provide short-term benefits, organizational-level interventions are more likely to generate sustainable, system-wide effects [11,47]. The S4R Toolbox therefore prioritizes strengthening organizational resilience as a structural pathway to improving individual resilience and mental well-being.

Evidence suggests that organizational resilience shapes how health care workers respond to stress and adapt to work-related challenges [48-50]. By targeting organizational structures, governance mechanisms, leadership practices, and coordination processes, the S4R intervention seeks to create enabling conditions that support both individual and collective resilience.

The Toolbox is designed to enhance leaders’ access to evidence-based knowledge about the needs of workers and informal caregivers, as well as organizational functioning. By facilitating informed leadership practices, it may strengthen resilience and promote supportive work environments. If effective, such changes could contribute to improved job satisfaction and workforce retention, which are critical challenges in older adult care.

### Contextual Adaptation Across Europe

Older adult care systems vary substantially across European countries [14]. Developing a tool applicable across diverse contexts requires balancing a stable, research-based core with adaptability to local conditions [37,51]. To address this challenge, the S4R project conducted early context mapping [14], applied a co-design approach involving empirical partners across countries [46], and incorporated continuous contextual updates throughout the development process.

While heterogeneity may limit direct cross-country comparability, it enhances ecological validity and allows evaluation of the Toolbox’s usefulness in diverse real-world settings. Evaluations will therefore primarily be conducted within countries, generating context-sensitive evidence regarding implementation feasibility, effectiveness, and cost-effectiveness, particularly in Norway and Finland.

### Evaluation and Policy Relevance

The S4R Toolbox will be evaluated through comprehensive process, effectiveness, and cost-effectiveness analyses. This multilevel evaluation approach is expected to provide robust evidence regarding implementation processes, outcomes, and economic implications across different European health systems, particularly in Norway and Finland. The resulting knowledge may provide actionable guidance for leaders, organizations, policy makers, and funders seeking strategies to strengthen resilience and mental well-being in older adult care.

### Strengths and Limitations

A key strength of the S4R project lies in its multicountry design, integration of theoretical frameworks, co-design methodology, and comprehensive evaluation strategy. Including diverse older adult care settings, such as nursing homes, home care services, and hospital-to-home transitions, allows for a more holistic understanding of resilience challenges in older adult care.

However, this heterogeneity also limits direct comparative analyses across countries and settings. Evaluations will therefore be conducted primarily within national contexts, which may limit broader generalizability.

Another limitation concerns the project time frame. Sustainable organizational transformation ideally requires long-term follow-up extending beyond 3-5 years. Funding constraints restrict the evaluation period, limiting conclusions regarding long-term sustainability.

### Future Directions

Future research should extend the integration of mental well-being, individual resilience, and organizational resilience to other health care contexts, including hospital systems and macro-level environments. Studies conducted outside Europe, including those in low- and middle-income countries, would further enhance generalizability and contextual understanding.

Longitudinal studies are needed to evaluate the sustainability and long-term cost-effectiveness of organizational resilience interventions. In addition, although S4R incorporates the perspectives of informal caregivers, future work should develop targeted tools to directly support the resilience and mental well-being of informal caregivers themselves.

Although the S4R project targets leaders as key agents of change, it does not directly intervene to strengthen leaders’ own resilience and mental well-being. Leadership roles in older adult care are often demanding and stressful, and leaders’ well-being significantly influences team functioning and staff outcomes [47,52]. Future research should therefore explore interventions specifically aimed at strengthening leaders’ resilience capacity.

### Conclusions

S4R will develop, implement, and evaluate a research-based Toolbox to support health care leaders in improving health care workers’ and informal caregivers’ resilience and mental well-being within older adult care. S4R will identify factors influencing resilience and mental well-being among health care workers and informal caregivers; explore their perspectives and needs; develop new theory on the relationships among individual resilience, organizational resilience, and mental well-being; and develop policy recommendations and cost-effective interventions. The S4R Toolbox, with tailor-made resources for policy and practical use, will be available through an open-access S4R Resource Bank. S4R will provide policy makers, decision makers, and health care leaders with solutions for taking action to address specific risks to health care workers’ and informal caregivers’ resilience and mental well-being. Thus, S4R will support the development of resilient health systems in older adult care through improved leadership capabilities, governance structures, and adaptive capacities. Results will be published in peer-reviewed journals, presented at international conferences, and disseminated through popular scientific platforms and social media.

## Appendix

Multimedia Appendix 1 Support4Resilience (S4R) questionnaires with response categories.

Multimedia Appendix 2 Overview of data collection.

Multimedia Appendix 3 Overview of intervention informants, activities, and outputs for tools 1, 2, and 3.

Multimedia Appendix 4 Intervention design.

Multimedia Appendix 5 Support4Resilience (S4R) Gantt chart.

## Abbreviations

CONSORT: Consolidated Standards of Reporting Trials
cRCT: cluster randomized controlled trial
EU: European Union
GDPR: General Data Protection Regulation
ICC: intraclass correlation coefficient
MRC: Medical Research Council
RCS: Resilience Capacity Scale
S4R: Support4Resilience
SWEMWBS: Short Warwick-Edinburgh Mental Well-Being Scale
WELLBY: Wellbeing-Adjusted Life Year
WP: work package

## Data Availability

Quantitative data collected in the Support4Resilience (S4R) project will be published in journals, on the S4R website, and in the S4R Resource Bank. The S4R Toolbox will be made available in a GitHub repository upon completion of the project.
