# Supplementary material for: Strengthening Resilience and Mental Well-Being Through the Support4Resilience Toolbox for Leaders in Older Adult Care: Protocol for a Cross-Country Mixed Methods Study (Support4Resilience)
Source: JMIR Res Protoc. 2026 Jul 20;15:e73701. doi: 10.2196/73701 (PMC13434252; doi:10.2196/73701)
Supplement: Supplementary file 1 [file resprot_v15i1e73701_app1.docx]

Appendix 4: S4R questionnaires with response categories

**Table 1. Questionnaires, publication references and translations available or translated by the project researchers**

**Questionnaires and their response options**

Leaders: 61 items

A. **Short Warwick-Edinburgh Mental Wellbeing Scale** (SWEMWBS) 7 items
B. **Connor-Davidson Resilience Scale** (CD-RISC) 10 items
C. **Resilience Capacity Scale** (RCS) 33 items
D. **Copenhagen Burnout Inventory** (CPI) 7 items
E. **Intention to turnover** (MOAQ) 3 items
F. **Self-assessed health** (SAH) 1 item

Healthcare workers: 61 items

A. **Short Warwick-Edinburgh Mental Wellbeing Scale** (SWEMWBS)* 7 items
B. **Connor-Davidson Resilience Scale** (CD-RISC) 10 items
C. **Resilience Capacity Scale** (RCS) 33 items
D. **Copenhagen Burnout Inventory** (CPI) 7 items
E. **Intention to turnover** (MOAQ) 3 items
F. **Self-assessed health** (SAH) 1 item

Informal caregivers: 49 items

A. **Short Warwick-Edinburgh Mental Wellbeing Scale** (SWEMWBS) 7 items
B. **Connor-Davidson Resilience Scale** (CD-RISC) 10 items
C. **Family Involvement in Care Questionnaire (FICQ)** 13 items
D. **Caregiver Self-Assessment Questionnaire** 18 items
E. **Self-assessed health** (SAH) 1 item

***Short Warwick-Edinburgh Mental Wellbeing Scale** (SWEMWBS) used for cRCT in NO and FI

**---------------------------------------------------------------------------------------------------------**

**Characteristics**

**1. Age**

**2.Gender**

1. Male
2. Female
3. Other

**3.What is your education (all groups)?**

1. PhD
2. Master’s degree
3. Bachelor/ three years of higher education (university or similar)
4. Less than three years of higher education (university or similar)
5. Secondary school
6. Primary school
7. None

**4.Length of time provided support for relatives** **(Informal caregivers)**

1. Less than 1 year
2. 1 – 5 years
3. 6 – 10 years
4. 11 – 15 years
5. 16 – 20 years
6. 21 years or more

**5.How much time do you spend on a weekly basis with the person (s) you are caregiver for (informal caregivers)?**

1. 1-5 hours a week
2. 5-10 hours a week
3. 10-20 hours a week
4. 20-30 hours a week
5. Most of the week
6. Live together

**6.Length of work experience (as a healthcare worker for healthcare workers and as a leader for healthcare leaders)**

1. Less than 1 year
2. 1 – 5 years
3. 6 – 10 years
4. 11 – 15 years
5. 16 – 20 years
6. 21 years or more

A. **Short Warwick-Edinburgh Mental Wellbeing Scale** (SWEMWBS)

Unidimensional, 7 items, 5-point Likert scale

1. I’ve been feeling optimistic about the future
2. I’ve been feeling useful
3. I’ve been feeling relaxed
4. I’ve been dealing with problems well
5. I’ve been thinking clearly
6. I’ve been feeling close to other people
7. I’ve been able to make up my own mind about things

Response categories

1. All of the time
2. Often
3. Some of the time
4. Rarely
5. None of the time

**---------------------------------------------------------------------------------------------------------**

B. **Connor-Davidson Resilience Scale** (CD-RISC)

Unidimensional, 10 items, 5-point Likert scale

Instructions: For each item, please mark an "x" in the box below that best indicates how much you agree with the following statements as they apply to you over the last month. If a particular situation has not occurred recently, answer according to how you think you would have felt.

1. Able to adapt to change
2. Can deal with whatever comes
3. Tries to see humorous side of problems
4. Coping with stress can strengthen me
5. Tend to bounce back after illness or hardship
6. Can achieve goals despite obstacles
7. Can stay focused under pressure
8. Not easily discouraged by failure
9. Thinks of self as strong person
10. Can handle unpleasant feelings

Response categories

1. Not true at all
2. Rarely true
3. Sometimes true
4. Often true
5. True nearly all of the time

**---------------------------------------------------------------------------------------------------------**

C. **Resilience Capacity Scale** (RCS)

Multi-dimensional, 33 items, 5-point Likert scale

10 capacities (leaders, healthcare workers, NOT informal caregivers include some capacities)

ITEMS

1. During stressful situations, we cooperate and support each other
2. We work together to resolve problematic situations
3. We listen carefully to each other when trying to solve problems
4. We experiment with alternative ways we might accomplish our work
5. We adjust our approach/es to overcome obstacles
6. We develop innovative ways of working
7. We optimise the resources we have to accomplish our work.
8. We have risk management strategies for day-to-day tasks
9. We have risk management strategies for emerging or unexpected events
10. We have open discussions related to possible risks
11. We have a good mix of skills and knowledge needed to carry out our work
12. We have a good "map" of each other's talents and skills
13. We share our unique skills and knowledge across specialties
14. We have a nearly ideal 'mix' of staff - a diverse set of people who bring different perspectives and skills to our work
15. Local management quickly adapts when conditions affecting staff/patients arise unexpectedly
16. Local management effectively monitors, prioritises and manages issues as they happen
17. Local management optimises resources and staff strengths to achieve outcomes
18. In team discussions, everyone's opinion is taken into consideration
19. In this unit, we do not hesitate to speak out openly if we think differently about a situation or solution
20. Staff in this unit seek out and act on feedback to improve our work
21. There are key staff who play an important role in implementing new knowledge and practices at [this facility]
22. There are key staff who motivate me and others in a positive way
23. There are key staff who transfer knowledge among team members and across specialities
24. The learning environment at [this facility] allows us to gain important insights from what we do well
25. The learning environment at [this facility] encourages us to continually improve how we do our work
26. The learning environment at [this facility] provides us with the space and time to learn from each other
27. There is well-functioning collaboration between units/departments at [this facility]
28. The exchange of information between units / departments runs smoothly at [this facility]
29. At [this facility], patients experience good continuity of care
30. [This facility] has clear strategies, plans and procedures to perform our work
31. At [this facility], we have the tools and information we need to do a good job
32. We know when and how to get help to manage issues or problems at [this facility]
33. Staff know what is expected of them at [this facility]

Response categories

1. Strongly disagree
2. Disagree
3. Neutral
4. Agree
5. Strongly agree

**---------------------------------------------------------------------------------------------------------**

D. **Michigan Organizational Assessment Questionnaire. Intention to turnover** (MOAQ)

3 items, Likert Scale

1. I often think about quitting
2. I will probably look for a new job the next year
3. I will actively look for a new job in the next year.

Response categories

1. Strongly disagree
2. Disagree
3. Slightly disagree
4. Neither disagree nor agree
5. Slightly agree
6. Agree
7. Strongly agree

**---------------------------------------------------------------------------------------------------------**

E. **Copenhagen Burnout Inventory** (CPI) 7 items
1. Is your work emotionally exhausting?
2. Do you feel burned out because of your work?
3. Does your work frustrate you?
4. Do you feel worn out at the end of the working day?
5. Are you exhausted in the morning at the thought of another day at work?
6. Do you feel that every working hour is tiring for you?
7. Do you have enough energy for family and friends during leisure time (inverse scoring)?

Response categories

1. Never/almost never
2. Seldom
3. Sometimes
4. Often/To a high degree
5. Always/To a very high degree)

**---------------------------------------------------------------------------------------------------------**

F. **Family Involvement in Care Questionnaire (FICQ)**

13 items, 5-point Likert scale

1. I have the opportunity to ask questions about my family member’s illness/condition
2. I understand the information I receive regarding my family member’s illness/condition
3. I receive sufficient information regarding my family member’s care
4. I can participate in discussions about which examinations/treatments that should be done
5. I participate in discussions about the goal of my family member’s treatment
6. I would have liked to be more involved in planning my family member’s care
7. The staff treats me with respect
8. The staff responds to my needs/wishes
9. It is easy to get in touch with the staff when I feel the need
10. I’m well received by the staff
11. I feel confident in the staff
12. I receive the emotional support I need in my family member's care
13. I have the opportunity to help my family member with everyday chores he/she usually manages on his/her own (e.g. going to the toilet, shaving/brushing hair or helping with meals)

Response categories

1. Fully agree
2. Largely agree
3. Somewhat agree
4. Fully disagree
5. Not relevant
G. **Self-assessed health (SAH) 1 item**

1.In general, would you say your health is:

- Poor
- Fair
- Good
- Very good
- Excellent

H. **Caregiver Self-Assessment Questionnaire**
   18 items, Yes/No

During the past week or so, I have:

1. Had trouble keeping my mind on what I was doing

2. Felt that I couldn’t leave my relative alone

3. Had difficulty making decisions

4. Felt completely overwhelmed

5. Felt useful and needed

6. Felt lonely

7. Been upset that my relative has changed so much from his/her former self

8. Felt a loss of privacy and/or personal time

9. Been edgy or irritable

10. Had sleep disturbed because of caring for my relative

11. Had a crying spell(s)

12. Felt strained between work and family responsibilities

13. Had back pain

14. Felt ill (headaches, stomach problems or common cold)

15. Been satisfied with the support my family has given me

16. Found my relative’s living situation to be inconvenient or a barrier to care

17. On a scale of 1 to 10, with 1 being “not stressful” to 10 being “extremely stressful” please rate your current level of stress

18. On a scale of 1 1 to 10, with 1 being “very healthy” to 10 being “very ill”, please rate your current health compared to what it was this time last year

Response categories for 1-16

1.Yes

2.No

Response categories for 17 and 18: A number from 1 to 10.
