# Supplementary material for: Strengthening Resilience and Mental Well-Being Through the Support4Resilience Toolbox for Leaders in Older Adult Care: Protocol for a Cross-Country Mixed Methods Study (Support4Resilience)
Source: JMIR Res Protoc. 2026 Jul 20;15:e73701. doi: 10.2196/73701 (PMC13434252; doi:10.2196/73701)
Supplement: Supplementary file 2 [file resprot_v15i1e73701_app2.docx]

| **Type of data collection** | **Frontline leaders** | **Healthcare workers** | **Informal caregivers** | **Norway** | **Finland** | **Romania** | **Spain** | **Netherlands** | **Italy** | **Australia** |
| --- | --- | --- | --- | --- | --- | --- | --- | --- | --- | --- |
| WP 2 | **The aim for the data collection in WP2 is to map the status of elderly care across the 6 empirical countries. This information will be used in the development of the S4R toolbox, as well as for policy recommendations, management models, intervention design and theory development. Furthermore, the quantitative data from WP 2 of healthcare workers will be used in the pre to post effectiveness analysis in WP4.** | | | | | | | | | |
| Quantitative data collection in WP2 | **Survey (totalling 300 informants in Europe + Australia (MU)** | **Survey (totalling 1200 informants in Europe + Australia (MU)** | **Survey (totalling 1000 informants in Europe + Australia (MU)** | **X**  **30 clusters in total (NO+FI)** | **X**  **30 clusters in total (NO+FI)** | **X**  **250-500 (leaders, workers and informal caregivers)** | **X**  **250-500 (leaders, workers and informal caregivers)** | **X**  **250-500 (leaders, workers and informal caregivers)** | **X**  **250-500 (leaders, workers and informal caregivers)** | **X**  **250-500 (leaders, workers and informal caregivers)** |
| Qualitative data collection in WP2 | **3-5 focus group interviews** | **3-5 focus group interviews** | **10 individual interviews** | **X** | **X** | **X** | **X** | **X** | **X** | **X** |
| WP 3 | **The aim of the pilot test is to evaluate and provide feedback on content, technological infrastructure, accessibility and usability by the real users.** | | | | | | | | | |
| Pilot | **Pilot of S4R intervention.**  **2 groups of leaders** |  |  | **X**  **4 units** |  |  |  |  |  |  |
| WP 4 | **The aim of WP 4 is to evaluate the S4R toolbox intervention through process evaluation, effectiveness, and cost effectiveness analysis.**  **Process evaluation will be informed by: Interviews (leaders, healthcare workers and informal caregivers) involved in the intervention and observation of workshops (leaders). The S4R toolbox contributes to support leaders, how workers and informal caregivers experience being involved and whether capacity-demands challenges improve. Additionally, to record changes on resilience capacities.**  **Effectiveness evaluation (pre to post) will be informed by survey data from healthcare workers involved in the intervention combined with data of leaders and healthcare workers in WP 2. Aim: The outcomes are healthcare workers’ mental wellbeing (SWEMWBS), resilience capacities (RCS), individual resilience (CD_RISC), Burnout (CPI) and intention to turnover (MOAQ). An additional objective is to validate the Resilience Capacity Scale.**  **Cost-effectiveness evaluation will be informed by survey data from healthcare workers of the involved sites and compared with the control group. Furthermore, informal on personal wellbeing from the sites (e.g., HR-questionnaires, turnover, sick leave, disability pensions) will be included. Aim: Primary outcomes are Mental wellbeing (SWMWBS)** | | | | | | | | | |
| Quantitative data collection in WP4 |  | **Survey (healthcare workers at intervention sites)** |  | **X (cRCT)**  **30 clusters in total (NO+FI)** | **X (cRCT)**  **30 clusters in total (NO+FI)** | **X**  **Approximately 200 workers** | **X**  **Approximately 200 workers** | **X**  **Approximately 200 workers** | **X**  **Approximately 200 workers** | **X**  **Approximately 200 workers** |
| Qualitative data collection in WP4 | **3-5 focus group interviews** | **3-5 focus group interviews** | **10 individual interviews** | **X** | **X** | **X** | **X** | **X** | **X** | **X** |
| Observation in WP4 | **During 3 workshops** |  |  | **X** | **X** | **X** | **X** | **X** | **X** | **X** |
