# Supplementary material for: Strengthening Resilience and Mental Well-Being Through the Support4Resilience Toolbox for Leaders in Older Adult Care: Protocol for a Cross-Country Mixed Methods Study (Support4Resilience)
Source: JMIR Res Protoc. 2026 Jul 20;15:e73701. doi: 10.2196/73701 (PMC13434252; doi:10.2196/73701)
Supplement: Supplementary file 3 [file resprot_v15i1e73701_app3.docx]

Appendix 2. Overview of intervention informants, activities and outputs for Tool 1, 2, and 3.

|  | **Informants** | **Activities** | **Outputs** |
| --- | --- | --- | --- |
| **Tool 1: Awareness** | Intervention team and local learning collaboratives in each country. | **2-hour workshop** tailoring  the intervention process to context and explaining key factors for resilience and wellbeing. | **Frontline leaders:** Developing a written process plan for the intervention (who, when, where, and how). |
|  | Frontline leaders, workers, informal caregivers. | **20 min questionnaire** Individual responses by all informants. | **Automatically generated by tool:** overview of status based on the combination of stakeholder inputs. |
|  | Frontline leaders and healthcare workers. | **3x1hour Group discussions** Frontline leaders and workers discuss survey results through using the elements in the learning resource. | **Frontline leaders:** Decided which areas to go into depth in Tool 2. |
| **Tool 2: Understand** | Frontline leaders. | **10x10 min. individual training**  engaging with E-learning modules. | **Frontline leaders:** Action plan for group discussions. |
|  | Intervention team and  local learning collaboratives. | **2-hour Workshop** follow up  discussion and planning for actions in Tool 3. | **Frontline leaders:** Developing a concrete, written action plan for group discussions. |
|  | Frontline leaders and healthcare workers. | **4x1 hour group discussions between** frontline leaders and healthcare workers using the reflexive scenario module. | **Frontline leaders:** Key learning points based on discussions suggestions for practices to reframe. |
| **Tool 3: Reframe** | Intervention team and  local learning collaboratives. | **2-hour Workshop** follow up  discussion and planning for next iteration. | **Frontline leaders:** Developing a concrete, written action plan for monthly meetings. |
|  | Frontline leaders and senior management. | **6x Monthly meetings** Discussions related to practices to keep, de-implement or implement new. | **Frontline leaders:** Consensus related to action plans and solutions for reframing practice. |
| **Evaluation:** Following the work with each tool, interviews of informants will be performed as part of qualitative process evaluation. In addition, members from the intervention teams and the local learning collaboratives in each country will attend one-hour evaluation sessions where the intervention will be evaluated. | | | |
