# Supplementary material for: Strengthening Resilience and Mental Well-Being Through the Support4Resilience Toolbox for Leaders in Older Adult Care: Protocol for a Cross-Country Mixed Methods Study (Support4Resilience)
Source: JMIR Res Protoc. 2026 Jul 20;15:e73701. doi: 10.2196/73701 (PMC13434252; doi:10.2196/73701)
Supplement: Supplementary file 4 [file resprot_v15i1e73701_app5.docx]

Appendix 4. S4R Gannt Chart
